# Intestinal Perforation: A Rare Complication of Treatment With Bevacizumab

**DOI:** 10.7759/cureus.14093

**Published:** 2021-03-24

**Authors:** Ramesh Adhikari, Medha Ghose, Aysun Tekin, Simranjit Singh, Romil Singh

**Affiliations:** 1 Hospital Medicine, Franciscan Health, Lafayette, USA; 2 Geriatrics, Brown University, Providence, USA; 3 Internal Medicine, Sir Salimullah Medical College, Dhaka, BGD; 4 Anesthesia Clinical Research Unit, Mayo Clinic, Rochester, USA; 5 Internal Medicine, Indiana University School of Medicine, Indianapolis, USA; 6 Critical Care, Mayo Clinic, Rochester, USA

**Keywords:** bevacizumab, perforation, cancer

## Abstract

Bevacizumab, a monoclonal immunoglobulin-G1 antibody directed against vascular endothelial growth factor (VEGF), inhibits angiogenesis. Gastrointestinal perforation is a serious and often fatal adverse event related to bevacizumab use. Bevacizumab is indicated in the treatment of colorectal malignancies, certain subtypes of non-small cell lung carcinoma, metastatic renal cell carcinomas, and cervical cancers. It is also indicated in the treatment of recurrent glioblastoma (GBM) in adult patients as the sole treatment agent or in combination with other antineoplastic medications. We present a case of a patient on bevacizumab currently with glioblastoma multiforme and seizures, who was previously treated with radiation treatment and temozolomide. The patient presented to the emergency room with abdominal pain, seizures and was diagnosed to have an intestinal perforation.

## Introduction

Vascular endothelial growth factor (VEGF), a diffusible glycoprotein produced by normal and neoplastic cells, is an important regulator of physiologic and pathologic angiogenesis [1]. Since angiogenesis is a crucial part of neoplastic growth, different mediators such as proteins and receptors such as VEGF and VEGF receptor (VEGFR) are important targets for anticancer treatments. Bevacizumab, a monoclonal antibody, works as a chimeric VEGF receptor, blocking VEGF and preventing it from binding to VEGFR [2].

In February 2004, the United States Food and Drug Administration (USFDA) approved bevacizumab as first-line therapy for metastatic colorectal carcinoma in combination with other chemotherapeutic drugs. It later gained traction as a first-line combination treatment for advanced metastatic non-squamous cell lung cancer. In 2009, bevacizumab was also approved as a treatment for glioblastoma multiforme (GBM) and renal cell carcinoma as a second-line treatment. Since then, several randomized clinical trials have shown that it improves survival, making it one of the most widely used oncologic drugs [3].

Like other chemotherapeutic agents, there are numerous adverse effects related to bevacizumab treatment such as hypertension, nausea, anorexia, diarrhea, electrolyte disorders, thrombocytopenia, and thromboembolism. Additionally, gastrointestinal perforation was documented in 0.3 to 3% of subjects in clinical trials [2]. We hereby report a fatal case of bowel perforation, occurring after bevacizumab administration in patients with GBM. 

## Case presentation

A 59-year-old female patient presented to the emergency department with the new onset of seizures and abdominal pain. Her past medical history was significant for essential hypertension. She was diagnosed with GBM 11 months ago while being evaluated for seizures. She received six weeks of low-dose radiation with concomitant temozolomide administration (115mg/day oral daily), which was discontinued due to refractory pancytopenia. Subsequently, she was initiated on bevacizumab treatment (10mg/kg IV every two weeks) two months ago.

The patient complained of significant abdominal pain a night before the presentation, as per her husband. On her way to the hospital, the patient had three seizure episodes and was treated with benzodiazepines in the emergency room. A review of systems couldn’t be done because of the patient’s postictal status. On the physical exam, the patient had abdominal tenderness with distention, rigidity, and guarding alongside neurological findings. 

Labs showed normal complete blood count levels with a basic metabolic panel (BMP) which was significant for low bicarbonate (19 mEq/L, normal range: 23-29 mEq/L) and mild elevation of high sensitivity troponin I (71 pg/mL, normal range: 0-12 pg/mL).

**Figure 1:**
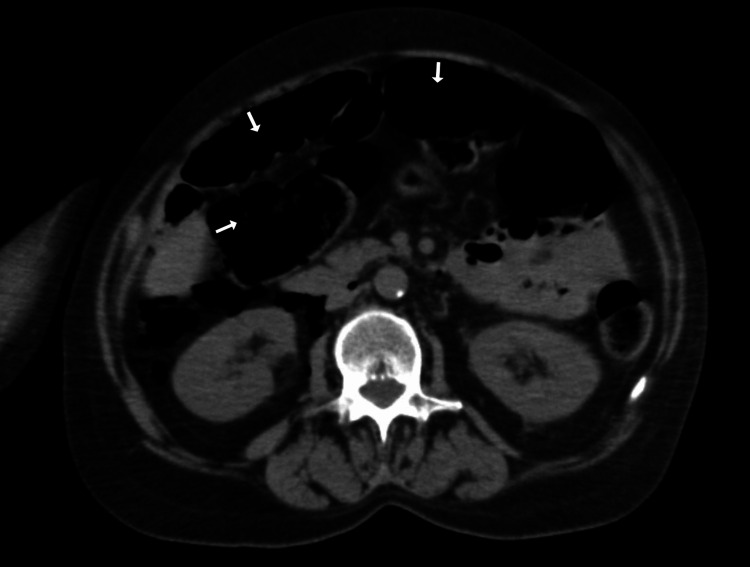
CT Scan Abdomen and Pelvis without contrast- Cross-sectional view with air in the peritoneum from Intestinal perforation.

**Figure 2:**
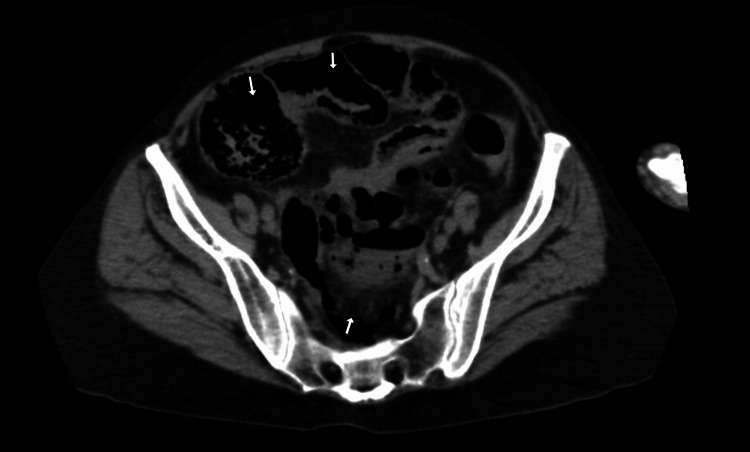
CT Scan Abdomen and Pelvis without contrast - Cross-sectional view- Air in the peritoneum due to intestinal perforation

**Figure 3:**
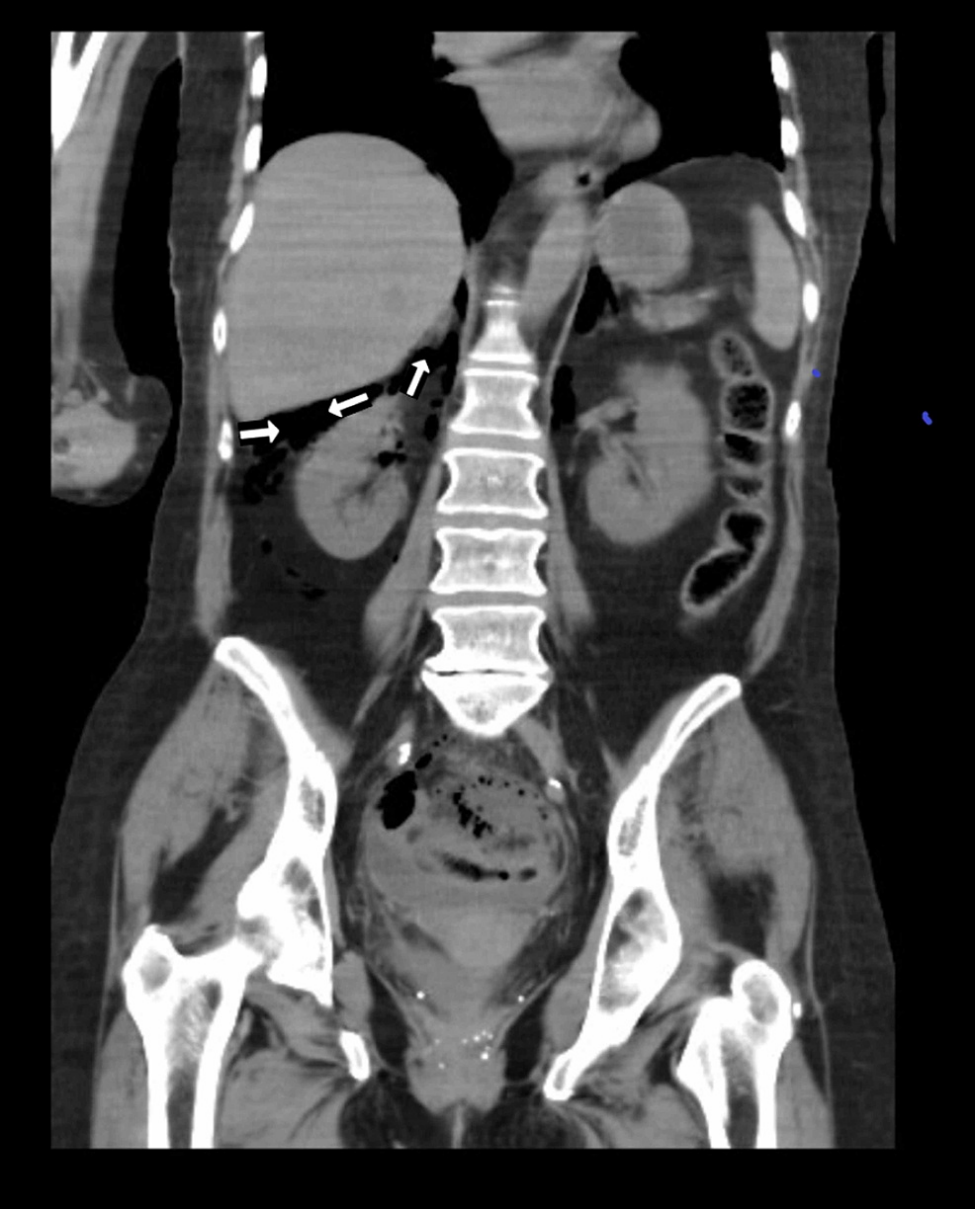
CT Scan Abdomen and Pelvis without contrast- Coronal View- Showing air under the liver from intestinal perforation.

**Figure 4:**
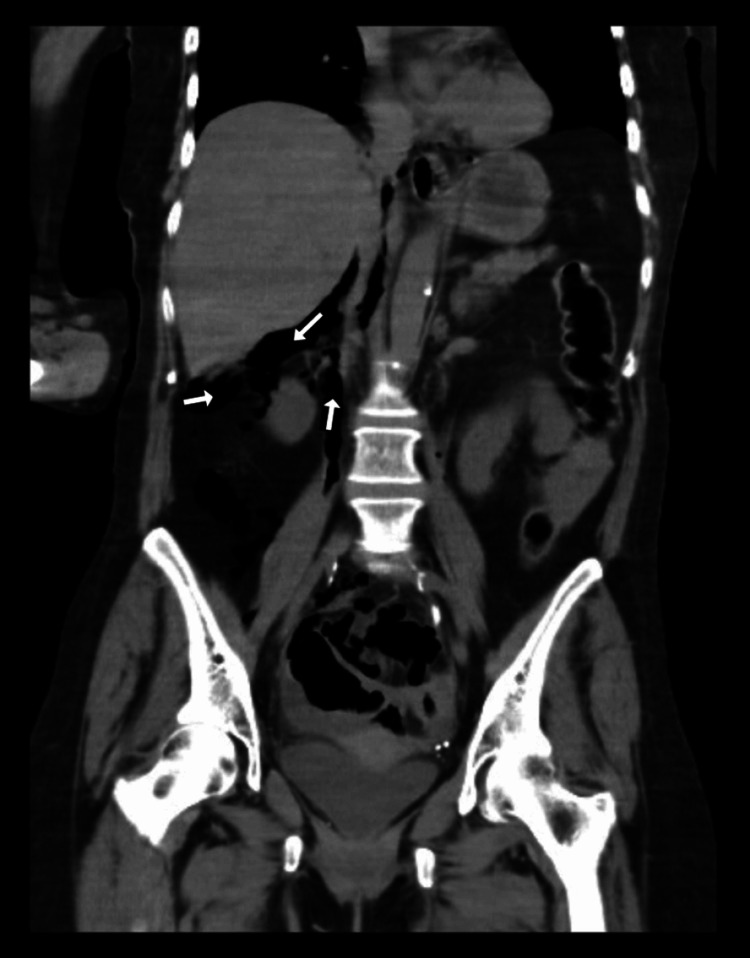
CT Scan Abdomen and Pelvis without contrast - Coronal View- Showing Air under the liver and Peritoneum from intestinal perforation.

On imaging studies, computerized tomography (CT) scan of the head showed the right frontal and temporal lobe masses with a stable subfalcine shift of 7.0 mm. No evidence of hemorrhage or obstructive hydrocephalus was found. The stat CT imaging of the abdomen revealed a large amount of retroperitoneal air, and some intraperitoneal air extending to the mediastinum. CT abdomen also showed distal colonic perforation with stool material outside of the colon in the pelvis and a sizable pelvic fluid collection was suspected. Colonic perforation was suspected to be at the descending colon/sigmoid colon junction, where the colon appeared somewhat thickened (Figures 1-4). An old CT scan of the abdomen showed a large diverticulum at the site of perforation. 

A stat consultation with the surgical team was obtained, and the condition of the patient was discussed with the patient's oncologist and husband. As per the patient’s wishes, comfort care was initiated. The patient eventually succumbed in the hospital couple of days into hospitalization.

## Discussion

As per the World Health Organization (WHO) classification GBM, or grade IV glioma, is the most common primary brain tumor in adults [4]. Standard treatment consists of maximal surgical resection followed by radiotherapy (RT) with concomitant and adjuvant temozolomide [5]. Median overall survival (OS) for patients with GBM is 14-15 months and five-year survival after diagnosis is less than 10% [6]. After RT and temozolomide, if there is a relapse or progression, there is no standard treatment and OS further decreases to three to nine months. The median progression-free survival is estimated at ten weeks, and the radiological response rate is usually less than 4%-16% [7, 8].

As one of the most vascularized human tumors [4], GBM produces proangiogenic factors, including VEGF which activates a downstream signal that results in angiogenesis, increases vascular permeability, and lymphangiogenesis by binding with their corresponding tyrosine kinase receptors [9].

Bevacizumab, a monoclonal antibody, works as a chimeric VEGF receptor [2]. It is approved for use in multiple cancers, including colon, lung, kidney, and cervix, as an individual treatment or as part of combination chemotherapy. Bevacizumab has been in clinical use since 2003 [10]. It was approved for use in recurrent GBM in 2009 [11, 12]. Recently bevacizumab was also USFDA certified for the treatment of liver cancer as adjunctive chemotherapy [13]. Drug interactions between bevacizumab and other agents used in the treatment of hepatitis B and C have been noted and therefore it is important to watch for drug-drug interactions, specifically in patients suffering from hepatitis [14].

The most common side effect related to bevacizumab use is hypertension. Other serious adverse events related to the treatment are proteinuria, hemorrhage, thromboembolism, arterial thromboembolic events, gastrointestinal perforation, poor wound healing, and reversible posterior leukoencephalopathy syndrome [2].

Gastrointestinal perforation

In clinical trials, 0.3 to 3% of patients receiving bevacizumab treatment-experienced gastrointestinal tract perforation [2]. Although underlying risk factors like bowel obstruction, chemotherapy-induced colitis, tumoral invasion of the gastrointestinal tract, previous treatments with abdominal irradiation, and bowel surgery have been shown to be related to gastrointestinal perforation with bevacizumab, it can also occur in patients without any risk factors [2].

In a phase II trial of bevacizumab alone or concomitant with irinotecan, bowel perforation occurred in 3% of the patients in the combined arm but did not occur in the patients given bevacizumab as a single agent [11].

The mortality rate of gastrointestinal perforation in patients treated with bevacizumab was reported as high as 50% [15]. The top three sites of gastrointestinal perforation are the colon, small intestine, and stomach, with the colon being the most common [16]. Several mechanisms for bevacizumab-induced perforation have been suggested, all of which are linked to microcirculation dysfunction caused by VEGF inhibition [4].

Out of 244 patients treated with antiangiogenic medications, six developed intestinal perforations in the study by Norden et al. As predisposing factors, one patient had a history of diverticulitis, while others had a history of corticosteroid use [17].

According to a single-arm phase II trial, the risk of bowel perforation is around 7.5 percent in patients with GBM who received bevacizumab combined with irinotecan and carboplatin, with one fatal case among them [15]. Patients receiving single-agent bevacizumab, on the other hand, showed no signs of bowel perforation in another study [11]. Intestinal perforation is more common in GBM than in other angiogenic neoplasms, according to current research. It's possible that it's linked to the use of corticosteroids in GBM [2].

Bevacizumab has indeed been linked to a number of wound-healing complications, including wound infection, ecchymosis, dehiscence, and surgical site bleeding. Patients must wait at least six to eight weeks after their last administration before having surgery. In addition, resuming bevacizumab after surgery should be delayed for at least 28 days to avoid a higher chance of wound healing complications, and a proper timeline should be followed to allow complete healing of the surgical wound. In all surgical patients, the negative effects of bevacizumab on wound healing must be addressed. This was a major reason why in our patient surgery was not considered due to the probability of poor wound healing [18]. 

The patient's symptoms are similar to those reported in the current literature as causing gastrointestinal perforation after bevacizumab treatment. The previous CT abdomen revealed a large diverticulum in this patient. The suggested treatment for acute patients is immediate resuscitation accompanied by an exploratory laparotomy. When patients are receiving bevacizumab, precautions should be taken to avoid any potential risk factors for gastrointestinal perforation, such as prophylactic procedures for patients with a history of diverticulitis or who are on corticosteroid therapy [2].

## Conclusions

Bowel perforation is a rare but potentially fatal complication of bevacizumab treatment. A high index of suspicion should be maintained while treating similar patients to avoid devastating outcomes. In patients presenting with acute abdominal pain after bevacizumab administration, no matter the duration of treatment, a prompt decision needs to be taken for early resuscitation and surgical intervention to increase the chance of survival. In chronic patients with risk factors, preventative management should be considered to prolong the life expectancy. 
